# Zinc-finger antiviral protein (ZAP) is a restriction factor for replication of modified vaccinia virus Ankara (MVA) in human cells

**DOI:** 10.1371/journal.ppat.1008845

**Published:** 2020-08-31

**Authors:** Chen Peng, Linda S. Wyatt, Shira G. Glushakow-Smith, Madhu Lal-Nag, Andrea S. Weisberg, Bernard Moss

**Affiliations:** 1 Laboratory of Viral Diseases, National Institute of Allergy and Infectious Diseases, National Institutes of Health, Bethesda, MD, United States of America; 2 Division of Preclinical Innovation, National Center for Advancing Translational Sciences, National Institutes of Health, Bethesda, MD, United States of America; University of Nebraska-Lincoln, UNITED STATES

## Abstract

Modified vaccinia virus Ankara (MVA) is an approved smallpox vaccine and a promising vaccine vector for other pathogens as well as for cancer therapeutics with more than 200 current or completed clinical trials. MVA was derived by passaging the parental Ankara vaccine virus hundreds of times in chick embryo fibroblasts during which it lost the ability to replicate in human and most other mammalian cells. Although this replication deficiency is an important safety feature, the genetic basis of the host restriction is not understood. Here, an unbiased human genome-wide RNAi screen in human A549 cells revealed that the zinc-finger antiviral protein (ZAP), previously shown to inhibit certain RNA viruses, is a host restriction factor for MVA, a DNA virus. Additional studies demonstrated enhanced MVA replication in several human cell lines following knockdown of ZAP. Furthermore, CRISPR-Cas9 knockout of ZAP in human A549 cells increased MVA replication and spread by more than one log but had no effect on a non-attenuated strain of vaccinia virus. The intact viral C16 protein, which had been disrupted in MVA, antagonized ZAP by binding and sequestering the protein in cytoplasmic punctate structures. Studies aimed at exploring the mechanism by which ZAP restricts MVA replication in the absence of C16 showed that knockout of ZAP had no discernible effect on viral DNA or individual mRNA or protein species as determined by droplet digital polymerase chain reaction, deep RNA sequencing and mass spectrometry, respectively. Instead, inactivation of ZAP reduced the number of aberrant, dense, spherical particles that typically form in MVA-infected human cells, suggesting that ZAP has a novel role in interfering with a late step in the assembly of infectious MVA virions in the absence of the C16 protein.

## Author summary

The attenuated vaccine vector known as modified vaccinia virus Ankara (MVA) was derived by extensively passaging the parental strain of vaccinia virus Ankara in chick embryo fibroblasts and is unable to replicate in most mammalian cells. The MVA host range restriction is exceptional in that synthesis of the abundant viral proteins appears unaffected but morphogenesis of virus particles is abortive. Despite the importance of the host range restriction for vaccine safety, the basis for this antiviral effect has remained an enigma. Here we demonstrate that the zinc finger antiviral protein (ZAP), previously shown to be an inhibitor of RNA viruses, is a specific host restriction factor for replication of MVA in human cells. Moreover, the intact vaccinia virus C16 protein, which was disrupted during the attenuation of MVA, sequesters ZAP in cytoplasmic punctae and effectively counteracts the inhibitory effects of ZAP.

## Introduction

During their co-evolution, viruses and their hosts developed antagonistic offensive and defensive strategies. The mechanisms employed are frequently species-specific and can become unbalanced if a virus infects a new host. In the laboratory, passaging a virus in cell culture under conditions in which viral defenses are superfluous may lead to spontaneous inactivation of some genes resulting in virus attenuation. The latter procedure forms the basis for the classical method of making safe vaccines as exemplified by the oral poliovirus vaccine. Another example of this method of attenuation is modified vaccinia virus Ankara (MVA), a licensed smallpox vaccine and a widely used vector for the development of vaccines against other pathogens [1, 2]. MVA was created in the 1960’s by passaging the parent chorioallantois vaccinia virus Ankara (CVA) hundreds of times in chick embryo fibroblasts (CEFs) leading to the inability of MVA to replicate efficiently in most mammalian cells. The MVA host range restriction is exceptional in that synthesis of the abundant viral proteins appears unaffected but morphogenesis of virus particles is arrested [3–5]. Indeed, the relatively high expression of recombinant proteins contributes to the success of MVA as a vaccine vector. Despite its importance for safety, the basis for the host range restriction of MVA has remained an enigma. Comparison of MVA with CVA reveals amino acid changes, insertions or deletions in 124 of 195 open reading frames (ORFs) [6].

We have taken a two-pronged approach to elucidate the host range defect of MVA. For the first tactic, we demonstrated that restoration of the inactivated viral genes encoding the C12 and C16 proteins enhanced replication of MVA in human cells [7, 8]. (To avoid misunderstanding, the VACV WR strain protein called C16 in several papers [9–11] corresponds to C10 according to the standard Copenhagen nomenclature and is entirely different from the MVA C16 host range protein [8]). C12 alone permits replication in MRC-5 cells but has little effect in other human cell lines, whereas C16 alone enhances replication moderately in all cell lines tested. Addition of the genes encoding both C12 and C16 to MVA fully reverses the human host range defect. Since MVA is competent to replicate in CEFs, this finding implies the existence of human host factors that are antagonized by C12 and C16. The second approach, described here, consisted of an unbiased human genome-wide RNAi screen to assess the effect of knockdown of individual host mRNAs on enhancing the spread of MVA. Knockdown or knockout (KO) of zinc-finger antiviral protein (ZC3HAV1), known as ZAP, was found to enable replication and spread of MVA in human cells.

ZAP was discovered as an antiviral protein that inhibits replication of Maloney murine leukemia virus by reducing the amount of cytoplasmic viral RNA [12]. Subsequent studies demonstrated that endogenous or overexpressed ZAP also inhibits alphaviruses [13], filoviruses [14], HIV-1 [15], hepatitis B virus [16] and influenza virus [17, 18], while other viruses including yellow fever virus, poliovirus, vesicular stomatitis virus and herpes simplex virus are resistant [13]. ZAP also has been reported to regulate murine gamma herpesvirus latency [19]. There are two main isoforms of ZAP: the long form (ZAP-L) has 902 amino acids and contains an N-terminal zinc finger RNA-binding domain, an internal TPH (trichohyalin-plectin-homology) domain, a WWE (Trp, Trp, Glu) domain and a catalytically inactive C-terminal PARP (poly ADP-ribose polymerase) domain; the short form of ZAP (ZAP-S) lacks the PARP domain [20]. ZAP binds to incompletely defined regions of specific RNAs and promotes its degradation or inhibits its translation. In the case of HIV-1 RNA, ZAP binds to a region with high CpG density [21]. Further studies indicate that the location rather than the total number of CpG dinucleotides is important [22]. ZAP lacks intrinsic RNase activity but recruits the 5’ and 3’ RNA degradation machinery [15, 23–25]. The E3 ubiquitin ligase TRIM25 is important for ZAP activity possibly by increasing translation inhibition [26]. Whether viruses that are resistant to ZAP encode specific inhibitors of ZAP or lack ZAP recognition signals is largely unknown.

We show that the non-attenuated Western Reserve (WR) strain of vaccinia virus (VACV) is resistant to endogenous ZAP, whereas MVA is sensitive. Furthermore, the intact VACV C16 protein interacts with and prevents the antiviral activity of ZAP. The inability to identify viral mRNAs that are specifically degraded or inactivated by ZAP is consistent with the latter having a novel inhibitory effect on a late stage of MVA morphogenesis.

## Results

### ZAP is a restriction factor for MVA

**Fig 1:**
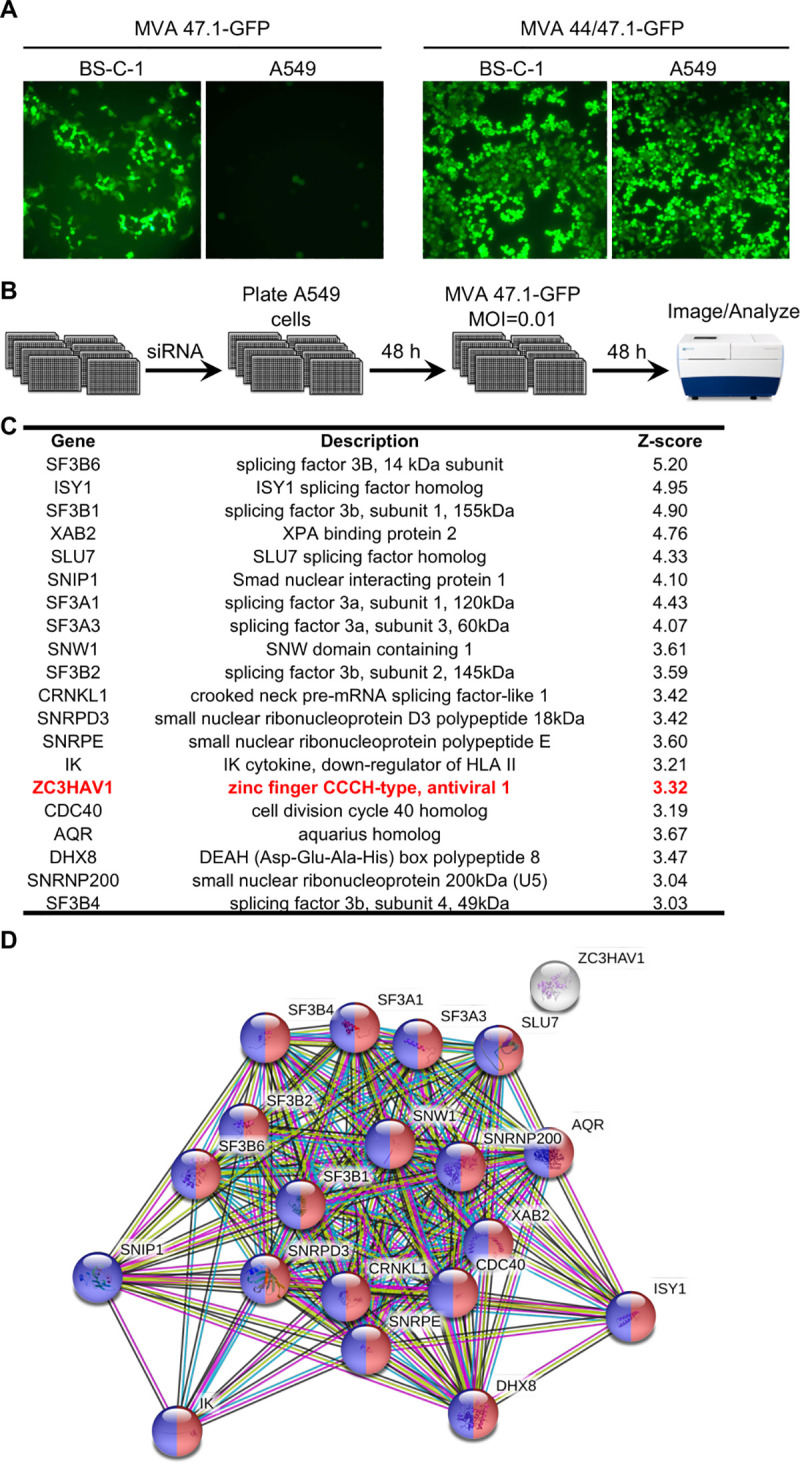
Identification of host restriction factors for MVA by human genome-wide RNAi screening. **(A)** A549 cells are non-permissive for MVA 47.1-GFP. BS-C-1 and A549 cells were infected with MVA 47.1-GFP or MVA44/47.1-GFP at 0.01 PFU/cell. Cells were imaged with a fluorescent microscope at 48 h post infection. (**B)** Outline of procedure for the genome-wide RNAi screen. MOI, multiplicity of infection. (**C)** Top 20 candidate genes identified from the screen and their corrected Z-scores. ZC3HAV1 is highlighted in red. (**D)** String analysis (https://string-db.org/) with candidate genes listed in (**C).** Red fill corresponds to GO term 398 (mRNA splicing via spliceosome); blue corresponds to GO term 6396 (RNA processing); white fill is ZC3HAV1.

Genome-wide RNAi is a powerful technique for identifying antiviral host factors. However, there are some caveats. First, since individual mRNAs are targeted, the method may fail if rescue depends on knockdown of more than one mRNA at the same time. Second, rescue may be only partial if more than one host factor contributes independently to the antiviral effect. Third, the effect may be indirect. To increase the chance of rescue we used the partial host range extended MVA 47.1, which recovered the ability to replicate in monkey BS-C-1 cells but not human cells [27]. In order to develop a high throughput assay, MVA 47.1 was modified by incorporation of the gene encoding enhanced green fluorescent protein (GFP) regulated by a VACV promoter into an intergenic site in the genome. The ability of MVA 47.1-GFP to spread in BS-C-1 but not in human A549 cells is shown in Fig 1A. For comparison, the fully host range extended MVA 44/47.1-GFP replicates in both cells because of recovery of disrupted viral genes [27](Fig 1A).

**Fig 2:**
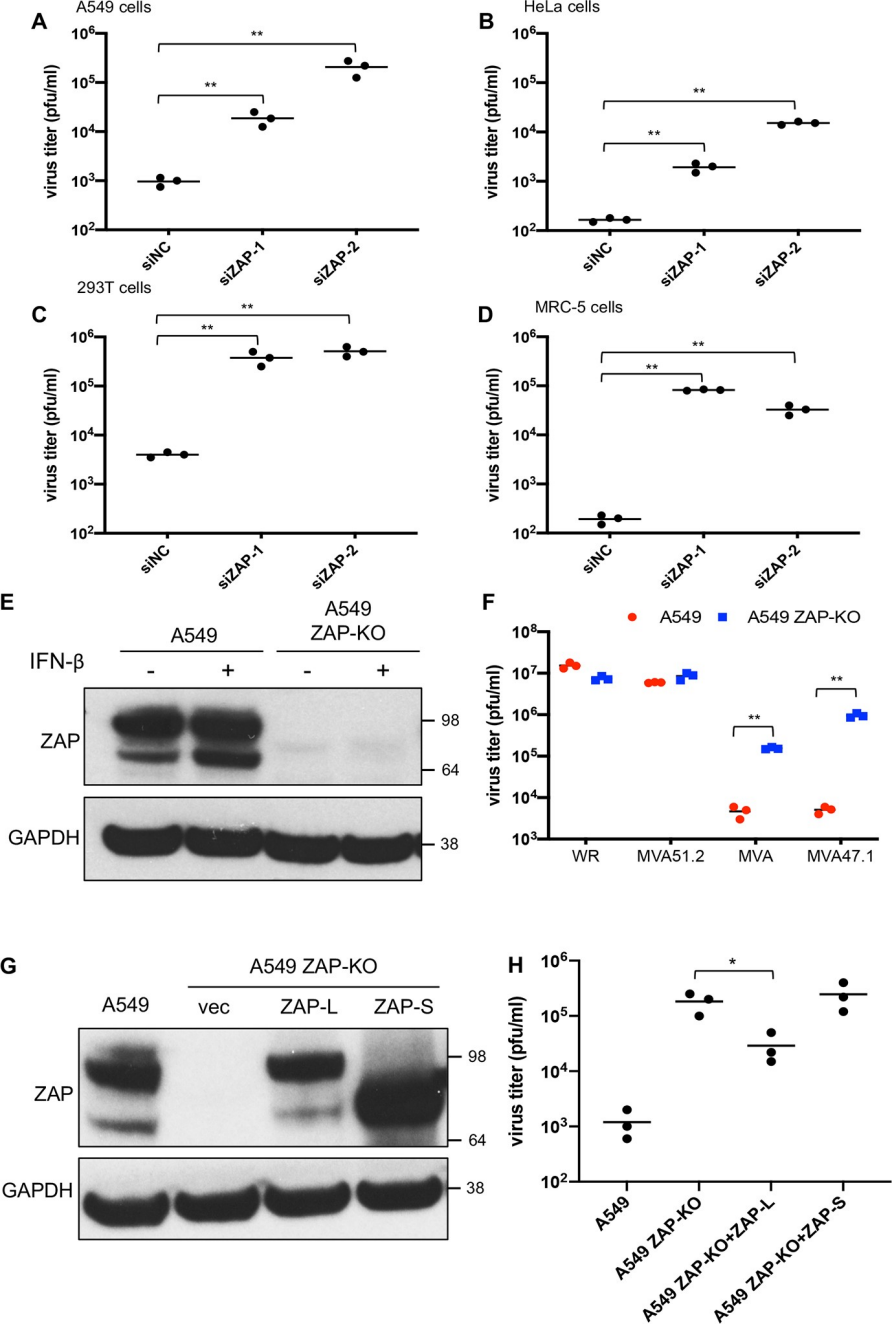
ZAP is a restriction factor for MVA in human cells. **(A-D)** Human A549, HeLa, 293T and MRC-5 cells were transfected in triplicate with siRNA for 48 h and then infected with MVA 47.1-GFP at 0.01 PFU/cell. Cells were collected after 48 h and virus titers determined by plaque assay on CEF. Virus titers from each infection are shown as dots, and the bar represents the mean value. siNC: non-targeting negative control siRNA. (**E**) A549 and A549 ZAP-KO cells were left untreated or treated with interferon-β for 24 h. Cell lysates were analyzed by SDS-PAGE and Western blotting with anti-ZAP antibody. GAPDH was used as a loading control. Numbers on the right represent the electrophoretic positions and masses of marker proteins in kDa. (**F**) A549 and A549 ZAP-KO cells were infected in triplicate with the indicated viruses at 0.01 PFU/cell. Cells were collected after 48 h and virus quantitated by plaque assay on CEF. Virus titers from each infection are shown as dots and the bar represents the mean value. (**G**) A549 cells and A549 ZAP-KO cells stably expressing ZAP-L, ZAP-S or vector control (vec) were lysed and proteins resolved by SDS-PAGE and Western blotting with anti-ZAP antibody. (**H**) Cell lines described in **G** were infected with MVA 47.1-GFP at 0.01 PFU/cell and cells were harvested at 48 h and virus titers determined by plaque assay on CEF. Virus titers from each infection are shown as dots and the bar represents the mean value. Statistics: * p<0.05; ** p<0.01 by two-sided Student’s t-test.

The basic RNAi protocol is outlined in Fig 1B. Three non-overlapping siRNAs for >20,000 human genes were individually reverse transfected into A549 cells in 384-well plates, which were then infected with a low multiplicity of MVA 47.1-GFP. After 48 h, the number of GFP positive cells were determined by automated fluorescence microscopy. The complete data set is provided in S1 Table and the subset with seed corrected Z-scores greater than three standard deviations from the mean is shown in Fig 1C. Of the 20 highest ranking genes, 18 were in Gene Ontology (GO) category 398 (mRNA splicing via spliceosome) and 19 in category 6396 (RNA processing) with only a single outlier (Fig 1D). Because VACV mRNA is synthesized in the cytoplasm and is unspliced, we thought it likely that the multiple nuclear RNA processing factors have indirect rather than direct effects on MVA replication. On the other hand, the outlier ZAP is a cytoplasmic protein with known antiviral properties, making it more likely than the others to directly inhibit MVA. To confirm the inhibitory role of ZAP, we tested the abilities of two additional siRNAs to knockdown ZAP in four human cell lines and enhance replication and spread of MVA 47.1 by determining virus titers rather than fluorescence. In A549, HeLa and 293T cells, the two main isoforms of ZAP (ZAP-L and ZAP-S) were expressed and knockdown of both were achieved with siRNAs (S1 Fig.). In MRC-5 cells, ZAP expression was lower than in other cells and ZAP-S was barely detected. Nevertheless, knockdown of ZAP also occurred in MRC-5 cells. Significantly increased virus titers were found when siRNA for ZAP was transfected into each of the human cell lines (Fig 2A–2D), confirming and extending the data from the RNAi screen.

Since ZAP mRNA is multiply spliced [33], we considered that the mRNA splicing and processing factors that were positive hits in the RNAi screen might reduce ZAP as well as other host mRNAs. However, two different siRNAs to ISY-1 and SF3A1 that were shown to knockdown their targets did not knockdown ZAP or enhance replication of MVA in A549 cells. Similar results were obtained when subconfluent and confluent cells were tested. Differences in the metabolic state of the A549 cells under conditions of large scale screening and small scale analysis may account for this discrepancy.

### ZAP-KO A549 cells support MVA replication

The permanent KO of a gene can have advantages relative to transient knockdown of mRNA expression. As a follow up to siRNA knockdown, CRISPR/Cas9 was used to inactivate ZAP in A549 cells. After transfection, cloned cells were screened by Sanger sequencing and by SDS-polyacrylamide gel electrophoresis (SDS-PAGE) and Western blotting. The latter confirmed the absence of ZAP-L and ZAP-S in the KO cells even in the presence of interferon-β, which increased expression of ZAP-S in unmodified A549 cells (Fig 2E). Importantly, replication and spread of MVA as well as MVA 47.1 was increased in the ZAP-KO cells compared to unmodified A549 cells, whereas the unrestricted WR strain of VACV and the host range extended MVA 51.2 replicated to similar extents in the two cell lines (Fig 2F). Thus, ZAP is a host range factor for unmodified MVA as well as MVA 47.1.

To further confirm the role of ZAP, we modified the ZAP-KO A549 cells to stably express either ZAP-L or ZAP-S regulated by the CMV promoter (Fig 2G). The ZAP-expressing cells were then infected with MVA 47.1. Reduction in virus titer occurred in cells transfected with the long but not the short isoform of ZAP even though the latter was more highly expressed (Fig 2H). Whether expression of ZAP-L and ZAP-S together would have a greater effect than ZAP-L alone was not evaluated.

### C16 but not C12 counteracts ZAP inhibition of MVA

**Fig 3:**
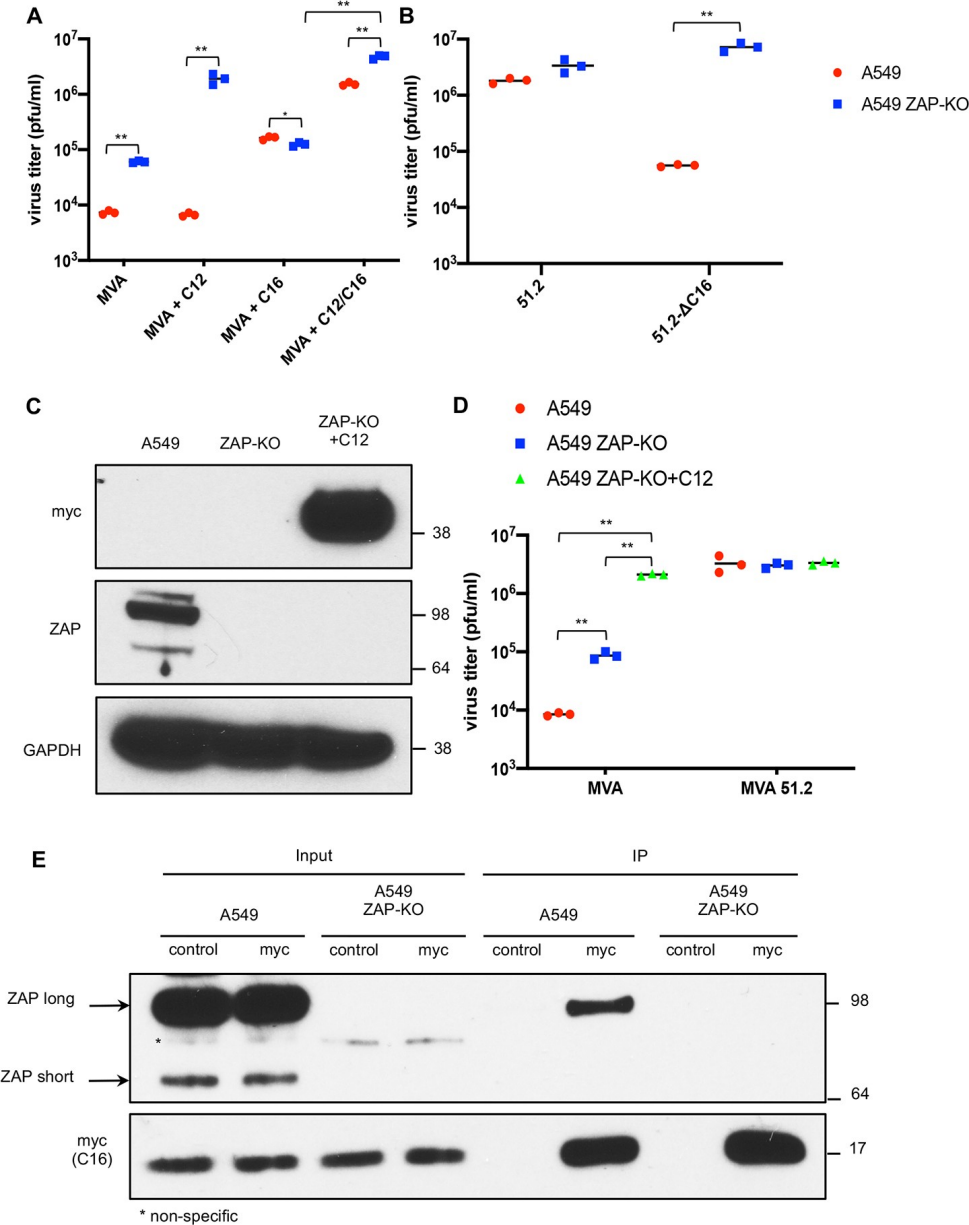
C16 antagonizes and interacts with ZAP. **(A**) A549 cells were infected in triplicate with MVA, MVA+C12, MVA+C16 or MVA+C12/C16 at 0.01 PFU/cell. After 48 h, the cells were harvested and virus titers determined by plaque assay on CEF. Virus titers from each infection are shown as dots and the bar represents the mean value. (**B**) A549 and A549 ZAP-KO cells were infected with 0.01 PFU/cell of MVA 51.2 or MVA 51.2-ΔC16 and virus titers shown as above. (**C**) Lysates from A549, A549 ZAP-KO and A549 ZAP-KO cells stably expressing C12 (ZAP-KO+C12) were analyzed by SDS-PAGE and Western blotting with antibodies to myc tag on C12, ZAP and GAPDH, which was a loading control. Numbers on the right represent the electrophoretic positions and masses in kDa of marker proteins. (**D**) The cells described in **C** were infected with MVA or MVA 51.2 at 0.01 PFU/cell for 48 h and virus titers were determined by plaque assay on CEF. Virus titers from each infection are shown as dots and the bar represents the mean value. **(E)** A549 and A549 ZAP-KO cells were infected with MVA-2xMyc-C16 at 10 PFU/cell for 18 h. Cell lysates were incubated with control magnetic beads or myc-trap magnetic beads (myc), extensively washed and proteins eluted with lithium dodecyl sulfate (LDS) buffer and analyzed by SDS-PAGE and Western blotting with antibodies to ZAP and myc epitope tag. Statistics: * p<0.05; ** p<0.01 by two-sided Student’s t-test.

We previously suggested that C12 and C16 proteins have different host targets because C16 increases MVA replication moderately in all human cells, whereas C12 is effective only in MRC-5 cells but together C12 and C16 fully overcome the host restriction [8]. Because of its activity in a broad range of cells, it seemed likely that C16 rather than C12 is a ZAP antagonist. To evaluate this hypothesis, we compared the abilities of MVA and recombinant MVAs expressing either C12 or C16 or both to replicate in unmodified A549 cells that express ZAP and A549 ZAP-KO cells that do not express ZAP. The prediction was that MVA expressing a ZAP antagonist would be relatively resistant to ZAP and would replicate to similar extents in A549 and A549 ZAP-KO cells. Indeed, this was the case: although the titer of MVA increased by more than a log in A549 ZAP-KO cells compared to A549 cells, the titers of MVA+C16 were similar (Fig 3A). In contrast, replication of MVA+C12 was not increased over MVA in A549 cells but was two logs higher in A549 ZAP-KO cells than in A549 cells, indicating that C12 alone is insufficient to inhibit ZAP (Fig 3A). The enhanced replication of MVA+C12/C16 compared to MVA+C12 or MVA+C16 in A549 cells was consistent with our previous study suggesting the two proteins have different targets [8]. The higher replication of MVA+C12/C16 in A549 ZAP-KO cells compared to A549 cells suggests that C16 may not always inactivate ZAP completely. It should be noted that only one of the two defective copies of C16 was replaced in MVA+C16 and that the parent CVA and other VACV strains such as Copenhagen have two intact copies, one in each inverted repetition.

The host range extended MVA 51.2 encodes both C12 and C16 and replicates well in A549 cells [27]. As another test of the role of C16, we infected unmodified A549 and A549 ZAP-KO cells with an MVA 51.2 mutant in which C16 was deleted but still expressed C12 (MVA 51.2-ΔC16). As predicted, replication of MVA-51.2 was similar in both cell lines whereas replication of the C16 deletion mutant was much higher in ZAP-KO A549 cells than in unmodified A549 cells (Fig 3B). Since C12 appears to have a target other than ZAP, we predicted that ZAP-KO cells engineered to express C12 under a CMV promoter would exhibit enhanced ability to propagate MVA. Western blotting confirmed stable expression of C12 with a myc epitope tag (Fig 3C). Moreover, replication of MVA in A549 ZAP-KO+C12 cells was similar to that of MVA 51.2, which encodes both C16 and C12 (Fig 3D).

Because FAM111A is a human host range restriction factor for a rabbitpox (RPXV) mutant deficient in C12 also called serine protease inhibitor 1 (SPI-1) [28], we considered that FAM111A might also contribute to the host range restriction of MVA. To test this possibility, we made a double KO A549 cell line in which both ZAP and FAM111A were inactivated as confirmed by a Western blot (S2A Fig.). Instead of enhancing replication of MVA, the titer was slightly reduced in the double KO cells compared to ZAP KO cells suggesting that FAM111A is not an additional restriction factor for MVA (S2B Fig.). To confirm the functional loss of FAM111A, the double KO cells were also infected with a RPXV C12 deletion mutant (RPXV-ΔC12) in which replication was greatly enhanced compared to unmodified and ZAP-KO A549 cells (S3B Fig.). Why replication of the RPXV mutant was reduced in ZAP-KO cells compared to unmodified A549 cells is not understood.

### Association of C16 and ZAP

To explore the basis for C16 antagonism of ZAP, we carried out experiments to determine whether ZAP is degraded or if the viral and host proteins interact. To evaluate the first possibility, A549 cells were infected with MVA 51.2, which expresses both C16 and C12 and overcomes the host restriction. However, Western blotting showed undiminished ZAP levels (S3 Fig.) indicating that degradation of ZAP had not occurred. To evaluate the second possibility, A549 and A549 ZAP-KO cells were infected with MVA+C16-myc. Proteins were captured on beads with antibody to the myc-tag attached to C16 and analyzed by Western blotting. The long isoform of ZAP was captured with C16-myc (Fig 3E). The short form was not detected even with longer exposure. Efforts to pull-down C16-myc with antibody to ZAP were unsuccessful and might require engineering an epitope tag on the endogenous ZAP protein.

**Fig 4:**
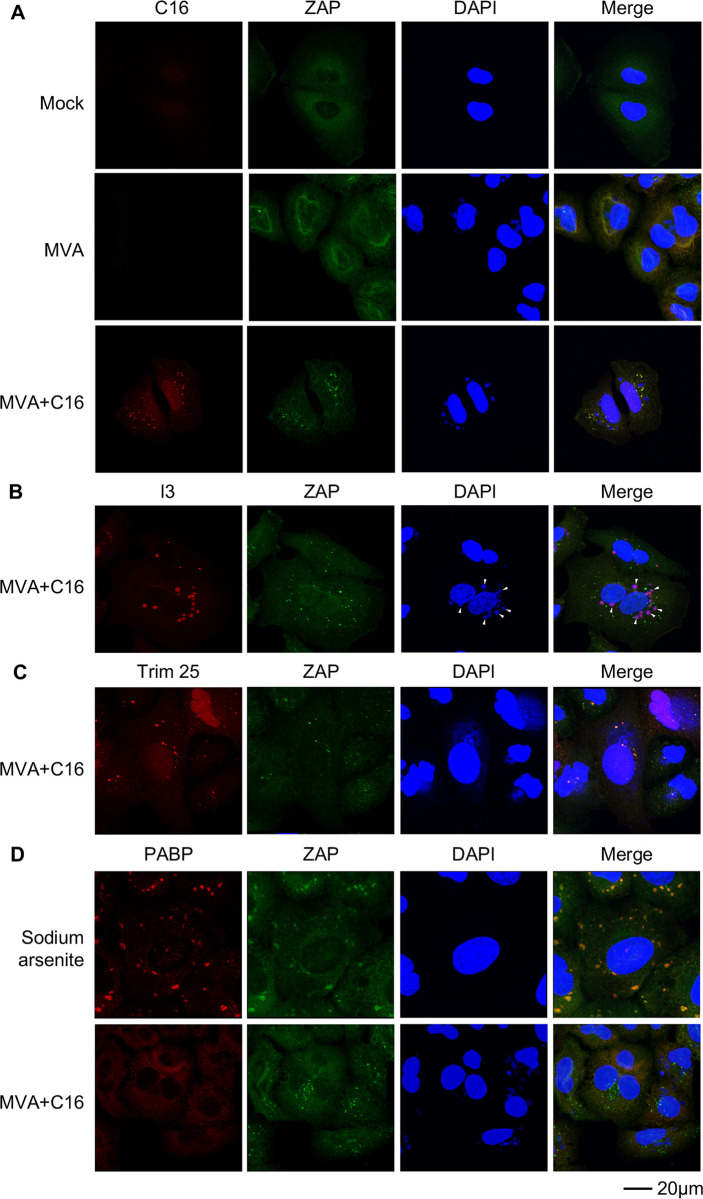
Co-localization of C16 and ZAP. **(A)** A549 cells were mock-infected, infected with MVA or MVA-2xMyc-C16 (MVA+C16) at 5 PFU/ cell for 5 h. Cells were then fixed, permeabilized, blocked and stained with primary antibodies to myc (C16) and ZAP followed by fluorescent conjugated secondary antibodies. DAPI was used to stain DNA. (**B**) A549 cells were infected with MVA-2xMyc-C16 and stained with antibodies to I3 and ZAP and with DAPI. Arrowheads point to virus factories. (**C**) A549 cells were infected with MVA-2xMyc-C16 and stained with antibodies to Trim25 and ZAP and with DAPI. (**D**) Uninfected A549 cells were treated with sodium arsenite or infected with MVA-2xMyc-C16 for 5 h and stained with antibodies to PABP and ZAP and with DAPI. Scale bar at bottom.

Confocal microscopy was used to determine the intracellular locations of C16 and ZAP as another method of assessing their interaction. In mock-infected cells, ZAP was diffusely distributed throughout the cytoplasm (Fig 4A). In MVA-infected cells in which C16 was not expressed, ZAP was still cytoplasmic but had a more perinuclear concentration (Fig 4A). In contrast, when cells were infected with MVA+C16-myc, ZAP formed punctate spots in the cytoplasm that co-localized with C16 (Fig 4A). The C16/ZAP punctae were not associated with DAPI-staining virus factories, nor did they co-localize with I3, a protein that does co-localize with factories (Fig 4B). In A549 ZAP-KO cells C16 was more diffusely distributed throughout the cell including the nucleus and formed fewer punctae (S4 Fig.) Thus, association of C16 with ZAP was shown by immunoaffinity purification and suggested by their intracellular co-localization in punctate bodies.

Previous studies had shown that TRIM25, an RNA-binding E3 ubiquitin ligase, enhances ZAP antiviral activity and that endogenous TRIM25 immunoprecipitates with ZAP [26, 29]. Using fluorescence confocal microscopy, we found that TRIM25 colocalized with ZAP punctae in cells infected with MVA+C16-myc (Fig 4C) indicating that C16 does not displace TRIM25 from ZAP. ZAP has been shown to associate with stress granules [30, 31], which we corroborated by treating uninfected A549 cells with sodium arsenite and localizing stress granules with poly(A) binding protein (Fig 4D). However, ZAP-C16 granules that formed after infection of A549 cells with MVA+C16 did not co-localize with poly(A) binding protein (PABP) (Fig 4D) nor with translation factors eIF4E nor eIF4G (S5 Fig.), which are also stress granule markers. Taken together our data show that C16 interacts with ZAP independent of stress granule formation and without preventing the interaction of ZAP and TRIM25.

### Viral genome replication and transcription in ZAP-expressing and ZAP-KO cells

**Fig 5:**
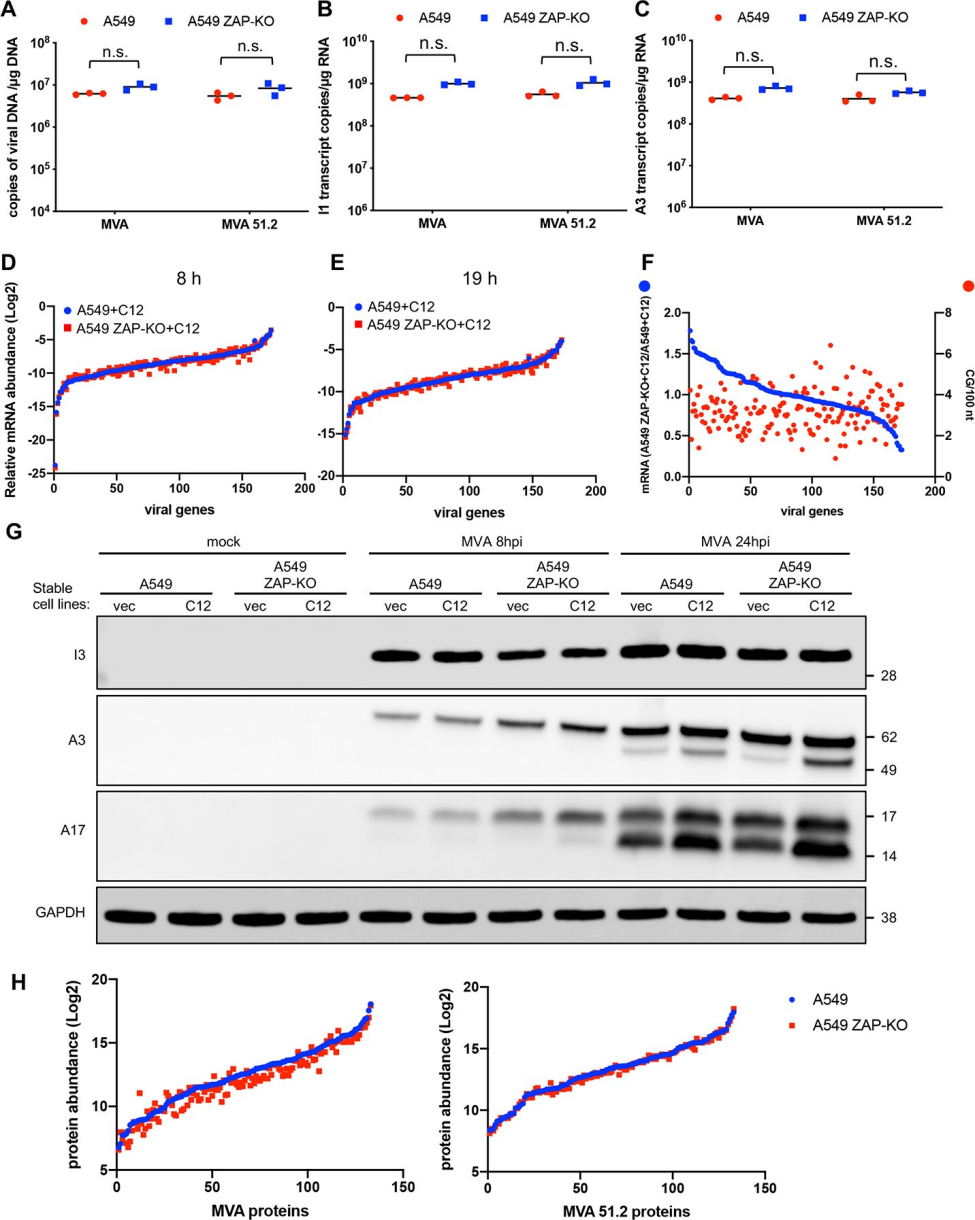
Effect of ZAP on MVA genome replication and gene expression. **(A)** A549 and A549 ZAP-KO cells were infected in triplicate with 5 PFU/cell of MVA or MVA 51.2. After 6 h, DNA was isolated and genome copies determined by ddPCR. DNA copies from each sample are shown as dots, and the bar represents the mean value. n.s., not significant, p>0.05. **(B, C)** A549 and A549 ZAP-KO cells were infected in triplicate with MVA or MVA 51.2 for 8 h. RNA was extracted, poly(A) selected, and cDNA prepared. Copy numbers of I1 and A3 transcripts per μg of RNA were determined with specific primers using ddPCR and shown as dots; the bar represents the mean value. (**D, E**) A549+C12 and A549 ZAP-KO+C12 cells were infected in triplicate as in above panels and RNA read counts at 8 and 19 h determined by RNAseq. Abundances of individual viral RNAs were plotted after normalization to total read counts in each sample. (**F**) Ratios of individual MVA mRNAs from A549 ZAP-KO+C12 cells/A549+C12 cells at 8 h after infection and CpG densities of individual mRNAs were plotted. (**G**) Expression and processing of viral proteins. A549, A549 ZAP-KO cells stably transfected with C12 or an empty vector (vec) were mock infected or infected with MVA at 5 PFU/cell. Total proteins were collected after 8 or 24 h and resolved by SDS-PAGE. Viral early I3, intermediate/late A3 virion core and A17 virion membrane proteins and GAPDH loading control were resolved by SDS-PAGE and detected by probing Western blots with specific antibodies. (**H**) Abundances of individual viral proteins from A549 and A549 ZAP-KO cells infected with MVA or MVA 51.2 for 18 h determined by TMT mass spectrometry.

Previous studies had concluded that viral protein synthesis is unimpaired in human cells infected with MVA, implying no significant effect on either genome replication or transcription [3]. However, neither viral DNA nor RNA synthesis was analyzed and only the most abundant viral proteins were detectable by the procedures used. Since ZAP is known to target specific RNAs, further analysis was warranted. First, we compared the amounts of viral DNA and representative RNAs in unmodified ZAP-expressing A549 cells and ZAP-KO A549 cells that were infected with MVA or the host range extended MVA 51.2, which replicates well in A549 cells. There was no significant difference in the number of viral genome copies in A549 and A549 ZAP-KO cells with either virus as determined by digital droplet PCR (ddPCR), although there was a trend to a slightly higher value in the ZAP-KO cells (Fig 5A). ddPCR was also used to compare the number of copies of intermediate and late I1 and A3 mRNAs in A549 and A549 ZAP-KO cells infected with MVA and MVA 51.2. No significant difference was found (Fig 5B and 5C).

The above comparison was limited to two abundant mRNAs. Deep sequencing was used for a more comprehensive analysis. The host range restriction of MVA is due to the absence of two proteins: C16, which we showed here antagonizes ZAP, and the C12 protein, which antagonizes a still unidentified host protein. In order to focus on the effect of ZAP specifically, we compared viral mRNAs in MVA-infected A549+C12 cells and A549 ZAP-KO+C12 cells. The complete data set is provided in S2 Table. For Fig 5D and 5E, the read counts of individual viral mRNAs were normalized to the total viral mRNAs in each sample. The data for the A549+C12 cells were plotted according to increasing read count. When the data from the A549 ZAP-KO+C12 were plotted in the same gene order, points closely traced those of the A549+C12, indicating no discernible pattern of enhancement by ZAP KO. A similar result was obtained when the early, intermediate and late classes of mRNA were graphed separately (S6 Fig.). In addition, the ratios of the MVA mRNA read counts in A549 ZAP-KO+C12 cells/A549+C12 cells had a near normal distribution varying from 0.5 to 1.5 (Fig 5F, blue line). Since there is evidence for interactions of ZAP with CpG-enriched regions of HIV mRNA, the ratios of the MVA mRNA read counts in A549 ZAP-KO+C12 cells/A549+C12 cells were plotted versus CpG-density (Fig 5F, red dots). The CpG-density did not correlate with a higher ratio of RNA read counts in A549 ZAP-KO+C12 cells/A549+C12 cells as would be expected if that were a factor in mRNA degradation (Fig 5F). Thus, no evidence was obtained for degradation of specific viral RNAs by ZAP.

### Analysis of viral proteins in A549 and A549 ZAP-KO cells

ZAP is an RNA binding protein that can inhibit translation as well as promote RNA degradation [32]. Further experiments were carried out to investigate the possibility of decreases in specific viral proteins in the presence of ZAP. Representative viral proteins were evaluated by infecting A549, A549+C12, A549 ZAP-KO and A549 ZAP-KO+C12 cells with MVA (Fig 5G) or MVA 47.1 (S7 Fig.). Viral early protein synthesis, as exemplified by I3, was similar in all four cell lines infected with MVA and MVA 47.1. The abundances of the intermediate/late core A3 and membrane A17 proteins were greater at 8 h in the ZAP-KO cell lines with or without C12 expression. However, by 24 h the amounts were similar in all cell lines. A3 and A17 undergo proteolytic processing as exemplified in the double bands in Fig 5G. At 24 h, the proteins had undergone proteolytic processing to similar extents in A549 and A549 ZAP-KO cells (Fig 5G, S7 Fig.).

Tandem mass tag labeling mass spectrometry was carried out to analyze a greater number of viral proteins. A549 and A549 ZAP-KO cells were infected with MVA or MVA 51.2 for 18 h. A total of 135 viral proteins were detected as shown in the full data set and the majority showed less than a 2-fold difference in the two cell lines (S3 Table). The similar abundances of the MVA and MVA 51.2 proteins from A549 and A549 ZAP-KO cells are shown in Fig 5H. There was more scatter in the MVA sample and further work is needed to evaluate the relevance of some small individual protein differences.

### Comparison of virion morphogenesis in A549 and A549 ZAP-KO cells

**Fig 6:**
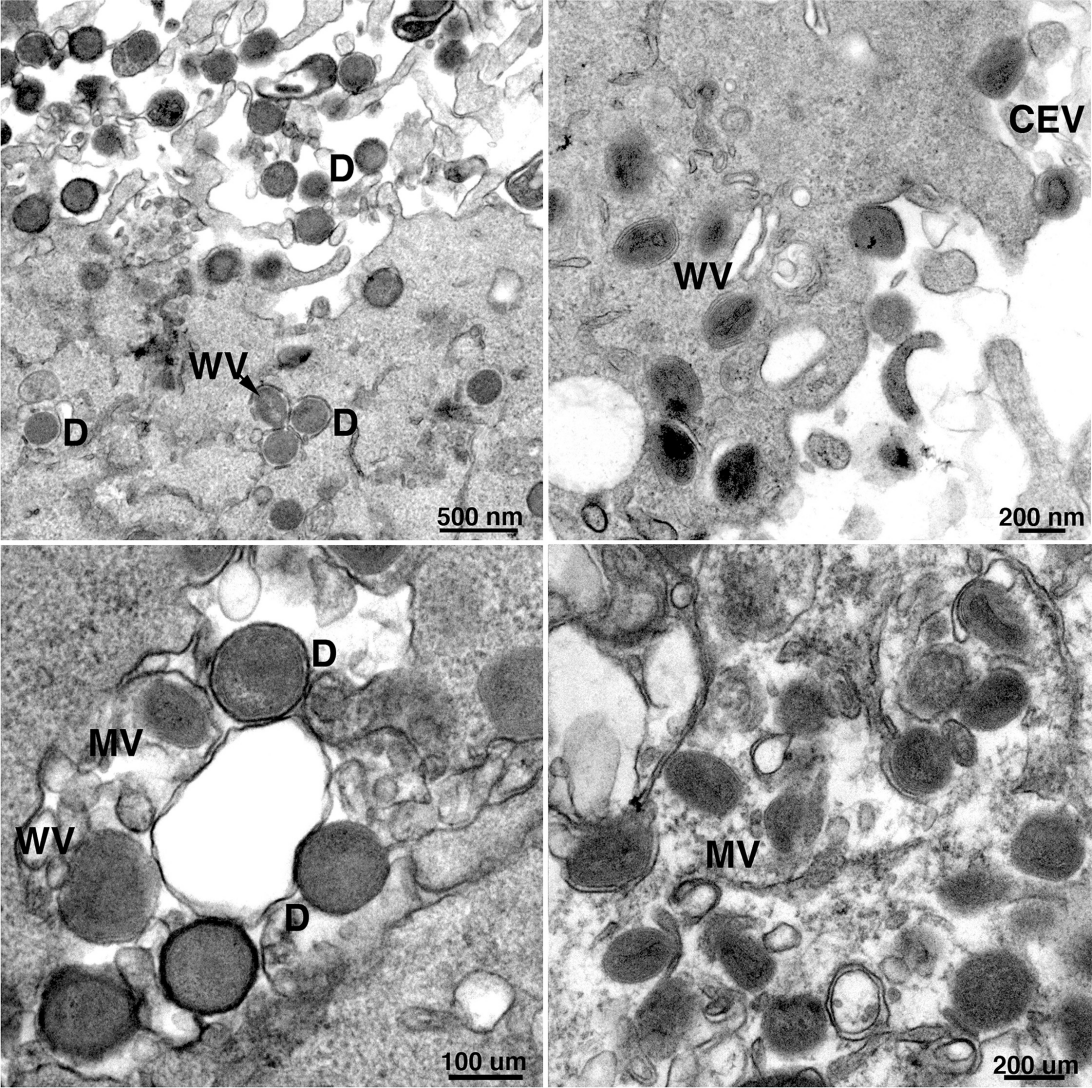
Transmission electron microscopy of cells infected with MVA. A549 cells (upper and lower left panels) and A549 ZAP-KO cells (upper and lower right panels) were infected with 10 PFU/cell of MVA. After 20 h, cells were fixed, sectioned and analyzed by transmission electron microscopy. Symbols: D, dense particle; MV, mature virion; WV, wrapped virion; CEV, cell-associated enveloped virion. Magnification factors shown below each panel.

VACV morphogenesis is a complex process starting with the formation of membrane crescents that extend to form spherical immature virions (IVs) enclosing core protein precursors and the viral genome. The IVs undergo a loss of the outer scaffold and condense to form barrel-shaped mature virions (MVs) with dumbbell-shaped cores that are infectious. Some MVs become enclosed by a double membrane derived from the trans-Golgi and endosomes to form wrapped virions (WVs) that are transported to the periphery of the cell where exocytosis occurs and extracellular virions (EVs) escape through the plasma membrane. EVs that adhere to the plasma membrane are referred to as cell-associated EVs or CEVs. A striking defect in MVA replication in human HeLa cells occurs during the late stage of morphogenesis. Transmission electron microscopy experiments showed elevated number of spherical immature and aberrant dense particles that lack a defined core structure [3–5]. We previously showed that the numbers of such defective particles were reduced in A549 cells infected with MVA expressing C16 [8]. If C16 is a direct antagonist of ZAP, then we would expect a similar effect on MVA morphogenesis by KO of ZAP. Indeed, few aberrant particles were observed in MVA-infected A549 ZAP-KO cells compared to A549 cells (Fig 6). Nearly 3-times more immature and 8-times more dense particles were found in 50 cell sections of MVA-infected A549 cells than in MVA-infected A549 ZAP-KO cells. Thus, inactivation of ZAP enhanced MVA morphogenesis in a similar manner as expression of C16.

## Discussion

The zinc-finger RNA binding protein ZAP was identified as a candidate restriction factor for MVA in an unbiased high-throughput human genome-wide RNAi screen in human A549 cells. The anti-viral role of endogenous ZAP was confimed by demonstrating that MVA replication was enhanced in four human cell lines by treating them with additional siRNAs to ZAP and in CRISPR-Cas9 ZAP-KO A549 cells. Additional studies suggested that ZAP-L, the long isoform of ZAP, which includes an inactive PARP domain, was inhibitory, though we did not exclude ZAP-S having an accessory role. Viruses differ in their susceptibility to the long and short isoforms of ZAP although both contain the N-terminal RNA binding domain [20, 33, 34]. In several cell lines including A549, the basal level of ZAP-L is higher than other isoforms, whereas ZAP-S is more highly induced by type 1 interferons [16, 35].

Having demonstrated that ZAP is a restriction factor for MVA, important questions remained: how do non-attenuated strains of VACV resist ZAP and how does ZAP inhibit MVA replication. Our previous studies showed that the host range restriction of MVA resulted from inactivation of the genes encoding the C12 and C16 proteins [7, 8]. Several lines of evidence implicated C16 as the ZAP antagonist. First, MVA replication was enhanced in several different cell lines by expression of C16 or knockdown of ZAP, whereas expression of C12 only enhanced MVA replication in MRC-5 cells. Second, KO of ZAP increased the replication of MVA expressing C12 but not C16 indicating that only the latter provided resistance to the antiviral effects of ZAP. C12 likely has another target because it enhances MVA replication in ZAP-KO cells. From a practical standpoint, ZAP-KO cells expressing C12 could be an alternative to CEF for propagation of MVA. Deletion of the C12 homolog in RPXV, also known as serine protease inhibitor I, causes a host range restriction in human cells that can be alleviated by KO of the host FAM111A and other proteins [28]. However, replication of MVA+C12 was not increased in ZAP/FAM111A double KO cells compared to ZAP-KO cells suggesting another target. An additional RNAi screen using A549 ZAP-KO cells instead of A549 cells might reveal the putative second host restriction factor for MVA.

Another question is how does C16 antagonize ZAP. One possibility was degradation of ZAP as mediated by the 3C protease of enterovirus A71 [36]. However, ZAP was not degraded during infection with a host range extended MVA expressing both C16 and C12; instead C16 was shown to interact with ZAP ostensibly preventing its antiviral activity. The interaction of C16 with ZAP was shown by co-immunoprecipitation and intracellular co-localization. In uninfected cells, endogenous ZAP had a diffuse cytoplasmic distribution, whereas in cells infected with MVA expressing C16, the latter protein co-localized in the cytoplasm with ZAP in punctate assemblies that lacked stress granule markers. TRIM25, an RNA-binding E3 ubiquitin ligase that interacts with ZAP and enhances the inhibition of Sindbis virus replication [26], was not a recognized hit in our RNAi screen. However, it could have been missed as the effect of TRM25 knockdown in the Sindbis virus system was 100-fold less than ZAP knockdown [26]. Nevertheless, TRIM25 localized with C16 and ZAP indicating that C16 does not displace TRIM25 from ZAP. C16 is conserved in many orthopoxviruses, although no role has been found with the exception of a host range factor for MVA. Bioinformatic analysis suggests that C16 has similarities to five other poxvirus BCL-2 proteins that function as immunomodulators [37, 38], two of which are also missing from MVA. Whether one of the other missing BCL-2 proteins can antagonize ZAP is under investigation.

Another important question is how ZAP inhibits MVA replication. Actually, the finding that ZAP is a host restriction factor for MVA was surprising because previous studies demonstrated inhibition of RNA viruses from several different families. Moreover, ZAP is an RNA-binding protein that has been shown to act by recruiting the mRNA decay apparatus or inhibiting mRNA translation [15, 23, 32, 39]. Another mode of action was described for the partial inhibition of influenza A virus replication by ZAP [17]. The ZAP-L PARP domain binds the PA and PB2 polymerase proteins, leading to their ubiquitination and proteasomal degradation. The latter antiviral activity is counteracted by the PB1 polymerase protein, which binds to an adjacent region of ZAP-L and causes PA and PB2 to dissociate from ZAP-L and thus escape degradation. Thus, the known mechanisms of ZAP involve specific RNA binding and decreased protein synthesis or degradation of specific proteins. On the other hand, the restriction of MVA replication in human cells is thought to involve an arrest in virion morphogenesis rather than inhibition of gene expression [3]. However, the conclusion that MVA gene expression is unaffected was based only on detection of the most abundant of the more than 150 proteins encoded by MVA. Since ZAP is known to bind specific RNAs, a plausible hypothesis was that the perturbation in morphogenesis results from diminished expression or degradation of one or few viral proteins. For this reason, it was important to more deeply analyze MVA mRNAs and proteins. We employed deep-sequencing of mRNA and mass spectrometry of proteins from MVA-infected ZAP positive and negative human cells. However, no distinct candidate target of ZAP was uncovered by the latter methods. ZAP has been shown to target RNAs with high density of CpG dinucleotides [21]. Nevertheless, genome-wide analysis failed to show CpG-rich regions in MVA and an inverse correlation of CpG density and mRNA abundance was not found. Nevertheless, fewer abnormal-looking virus particles were detected by electron microscopy in MVA-infected A549 ZAP-KO cells than in A549 cells consistent with an effect of ZAP on virion morphogenesis. Proteolytic processing of membrane and core proteins was not prevented by ZAP, suggesting an effect at a late stage in virion maturation. Whether ZAP directly perturbs morphogenesis by interacting with specific viral proteins remains to be determined.

## Materials and Methods

### Ethics statement

Experiments and procedures were approved under registration #7193 by the Division of Occupational Health and Safety, Office of Research Services, NIH.

### Cell culture

BS-C-1 (ATCC CCL-26) and MRC-5 (ATCC CCL-171) cells were maintained in Eagle’s Minimum Essential Medium (EMEM) containing 10% fetal bovine serum (FBS) supplemented with 100 units penicillin, 100 μg of streptomycin and 2 mM L-glutamine. 293T cells (ATCC CRL-3216) and HeLa cells (ATCC CCL-2) were grown in Dulbecco’s Modified Eagle Medium (DMEM), A549 cells (ATCC CCL-185) were grown in DMEM-F12 supplemented as above. Primary CEFs were prepared from 10-day-old fertile eggs (Charles River) and maintained in EMEM medium supplemented as described above.

### Virus infection and plaque assay

The culture medium was removed from cells at 90% confluency and replaced with virus inoculum in complete EMEM containing 2.5% FBS to reach a titer of 0.01 PFU/cell. After 2 h at 37°C, inoculum was removed, and cells were washed twice with PBS prior to adding fresh medium. Cells were collected and lysed after 48 h and virus titers were determined by plaque assay in CEF as described [40].

### Construction of recombinant MVAs

MVA 47.1+GFP was made by inserting the enhanced GFP ORF directed by a synthetic early/late promoter [41] into MVA between the F12 and F13 ORFs. Deletion of C16, and insertion of C12 and/or C16 were described previously [7, 8].

### Genome-wide RNAi screen

The Ambion Silencer Select Human Genome siRNA Library version 4 targeting 21,500 genes, most with three non-overlapping single siRNAs per gene, was used to reverse transfect human A549 cells in 384-well plates. After 48 h, cells were infected with 0.01 PFU/cell of MVA 47.1+GFP and 48 h later were fixed and stained with DAPI. Image processing was carried out with a Molecular Devices ImageXPress Micro Confocal high-content device as previously reported [42]. Genes were ranked by the percentage positive GFP cells. The RSA analysis tool was used to deprioritize potential false positives on the basis of off-target effects [43].

### siRNA transfection

siRNAs were designed with the IDT RNAi design tool (Integrated DNA Technologies) and chemically synthesized. siRNA (10 pmol per well) was diluted in opti-MEM (ThermoFisher) and mixed with RNAiMax transfection reagent (ThermoFisher) for 5 min at room temperature. The siRNA-transfection reagent mixture was added to and incubated with cells for 48 h prior to viral infection.

### Generation of ZAP-KO cell line with CRISPR-Cas9

Two different sgRNAs were designed to target the exon 1 region of human ZAP (5’-GGCCGGGATCACCCGATCGG-3’, 5’- GGATCACCCGATCGGTGGTG-3’) and were inserted into pSpCas9(BB)-2A-GFP, a gift from Feng Zhang (Addgene plasmid #48138; http://n2t/addgene_48138). A549 cells were transfected with the above plasmids at 60% confluency and sorted by flow cytometry based on the expression of GFP at 48 h post-transfection. GFP positive cells were serially diluted for clonal selection and successful inactivation of ZAP was determined by Sanger sequencing and Western blotting with a ZAP antibody (Proteintech).

### Generation of stable cell lines expressing human ZAP or VACV proteins

C12 and C16 ORFs from MVA 51.2 were codon-optimized for mammalian expression and chemically synthesized. ZAP-L and ZAP-S ORFs with N-terminal HA tags [20] were copied from plasmids received from Addgene (ZAP-L: #45907, ZAP-S: #45906) that were deposited by the Harmit Malik laboratory. The above sequences were then cloned into a retroviral vector pQC-XIP (Takara Bio.), which contains a puromycin resistant gene for antibiotic selection. Pseudoviruses were packaged in human 293T cells by co-transfection and filtered prior to transduction of A549 and A549 ZAP-KO cells in the presence of polybrene. Transduced cells were then passaged for 2 weeks in medium containing 1μg/ml of puromycin for selection. Expression of transgenes were confirmed by Western blotting.

### Western blotting and antibodies

Cells were washed once with ice-cold phosphate buffered saline (PBS) prior to protein extraction and total protein was collected with lithium dodecylsulfate (LDS) buffer supplemented with 0.05 M DTT and phosphatase/protease inhibitor cocktail (ThermoFisher). The protein samples were briefly sonicated to reduce viscosity, boiled at 95°C for 5 min and then resolved on 4–12% NuPAGE Bis-Tris gel (ThermoFisher). Protein was transferred to nitrocellulose membranes with an iBlot2 dry transfer system (ThermoFisher). Membranes were blocked in 5% non-fat milk in Tris buffered saline with 0.1% Tween 20 (TBST, Sigma) for 1 h at room temperature and incubated with primary antibodies at 4°C in blocking buffer overnight. On the second day, membranes were washed three times with TBST and incubated with secondary antibodies conjugated with HRP in blocking buffer for 1 h at room temperature. Protein was detected with SuperSignal West Dura substrate. Antibodies to VACV A17, A3 and I3 proteins [44, 45] were previously described; antibody to ZAP was purchased from Proteintech (16820-1-AP). Myc antibody conjugated to HRP was purchased from SantaCruz (9E10) and GAPDH antibody was from Cell Signaling Technology (CST, 2118S). Rabbit and mouse secondary antibodies conjugated to HRP were purchased from Cell Signaling Technology.

### Immunoprecipitation

A549 cells and A549 ZAP-KO cells in 10 cm dishes were grown to 90% confluency and infected with MVA-myc-C16 at 10 PFU/cell. After 18 h, cells were washed once with ice-cold PBS and lysed with 20 mM Tris-CL [pH 7.4], 150 mM NaCl, 2 mM EDTA, 1% Triton X-100 containing freshly added protease inhibitors (ThermoFisher) on ice for 30 min with frequent agitation. Cell lysates were centrifuged for 15 min at 15,000 x g at 4°C and then incubated with control magnetic beads or Myc-Trap magnetic beads at 4°C for 3 h with rotation. The beads were then wash 6 times with lysis buffer and the bound proteins were eluted by boiling with LDS sample buffer containing 0.05 M DTT for 15 min. The proteins were then resolved on 4–12% NuPAGE Bis-Tris gels and analyzed by Western blotting with antibodies to ZAP and myc epitope tag.

### Quantification of viral genomic DNA and viral mRNA by ddPCR

A549 and A549 ZAP-KO cells were infected with MVA or MVA 51.2 at 5 PFU/cell. Cells were washed twice with PBS and harvested for DNA or RNA extraction. Total DNA was isolated with DNeasy Blood/Tissue DNA mini kit (Qiagen) at 6 h post-infection and quantitated by nanodrop spectrophotometer (ThermoFisher). Total RNA was extracted at 8 h after infection with RNeasy RNA mini kit (Qiagen), quantitated with the nanodrop spectrophotometer and equal amounts of RNA were treated with DNase prior to reverse-transcription with a SuperScript IV First-Strand synthesis system using oligo-dT primers (ThermoFisher). Equal amounts of DNA or cDNA from each sample were then serially diluted and analyzed with gene-specific primers (E11L for viral genomic DNA) by ddPCR according to the protocol described previously using an automated droplet generator and the QX200 droplet reader (Bio-Rad) [46]. Primers used for mRNA quantification: I1L (5’ TGGAAAACTGGATGATACAGGCA 3’; 5’ TGTGTAGCGCTTCTTTTTAGTC 3’); A3L (5’ CTATAGACAAAATAGAAGCC 3’; 5’ CCATGATTAGAAAAGCAATTATG 3’). Data analysis was performed with QuantaLife software (Bio-Rad) that uses Poisson distribution statistics.

### RNA sequencing

Cells were harvested into Trizol, lysates combined with 1-bromo-3-chloropropane (Millipore Sigma, St. Louis, MO), and the RNA containing aqueous phase was collected from each sample and passed through Qiashredder column (Qiagen, Valencia, CA) to shear any remaining genomic DNA. RNA was extracted using Qiagen AllPrep DNA/RNA 96-well system (Valencia, CA). An additional on-column DNase 1 treatment was performed during RNA extraction. RNA integrity was assessed using the Agilent 2100 Bioanalyzer using RNA 6000 Pico kit (Agilent Technologies, Santa Clara, Ca). RNA integrity number (RIN) ranged from 6.8 to 9.6. One microgram of each sample was used to generate sequencing libraries using the TruSeq Stranded mRNA Sample Preparation Guide, Rev. E (Illumina Inc., San Diego, CA) using the low-throughput (LS) protocol and single-read indexes. Sequencing was carried out on an Illumina NextSeq 550 Sequencing System for 75 cycles in each read direction producing an average of 37 million reads per sample. Raw reads were trimmed of adapter sequence and low-quality bases and filtered for low quality reads using the FASTX-Toolkit (Hannon Lab, CSHL). Remaining reads were mapped to the VACV genome (U94848.1) using Bowtie2 [47]. Reads mapping to genes were counted using htseq-count [48]. Data normalization and differential expression analysis was performed using the Bioconductor package DESeq2 [49], with a padj less than 0.05 considered significant.

### Tandem mass tag labeling mass spectrometry

Pellets containing 1.2x10^7^ cells were lysed in 1 ml of buffer containing 50 mM HEPES, pH 8.5 and 6 M guanidinium HCl with vigorous mixing and then sonicated. Following centrifugation at 18,000 xg, 400 μg of supernatant protein was reduced with DTT and alkylated with iodoacetamide. Guanidinium HCl was added to 1.5 M in 50 mM HEPES, pH 8.5 followed by addition of 2 μg of rLysC protease (Promega) and incubation at 37°C for 6 h. The guanidinium HCl was diluted to 0.8 M by the addition of 100 mM HEPES, pH 8.0 and digestion was continued for 15 h after the addition of 4 μg trypsin. The pH was adjusted to 2.5 by the addition of trifluoroacetic acid (TFA) and the samples were desalted and concentrated using SepPak Light C_18_ cartridges. Elution was with a 0.1%TFA, 50% acetonitrile followed by evaporation under vacuum at 50°C. Peptides **(**100 μg) from each of the six samples were labeled with isobaric TMT-6plex, 126 to 131 (ThermoFisher) and samples were mixed together. Desalting was done on an Oasis HLB vacuum cartridge (Waters) and labeled peptides were reduced to dryness under vacuum The peptides were fractionated by alkaline reverse phase chromatography on a 0.46 mm x 250 mm C_18_ XBridge column equilibrated in 5 mM triethylammonium bicarbonate buffer pH 8.3, 3% acetonitrile and developed with a linear gradient to 50% acetonitrile over 130 min. 125 fractions were collected and mixed to create 20 fraction pools for analysis by nanoLCMS (Lumos Orbitrap mass spectrometer). The raw data files were processed using Proteome Discoverer v2.4.0.305 using a SEQUEST HT search against a concatenated database containing Human (Uniprot KB/Swiss-Prot; 04/2020) and MVA (U84848.1; Uniprot KB; 07/2019) proteins. For the searches a 10 ppm precursor mass tolerance and a 0.6 Da fragment mass tolerance were used with oxidation [M] and acetylation [protein N-term] as dynamic modifications and carbamydomethylation [C] and TMT 6plex [K/peptide N-term] as fixed modifications. The 1% peptide false discovery threshold was calculated using the percolator algorithm as implemented within Proteome Discoverer and a 2 peptide per protein, 1% protein FDR was required for inclusion in the report. Reporter ions were quantified at the MS3 order using a 20 ppm integration tolerance. For final quantitation values were corrected using the factors reported for lot number TG257173 (ThermoFisher Scientific) with a protein level rollup of unique and razor peptides for abundance calculations. Normalization was performed using all human proteins identified in the analysis.

### Confocal microscopy

A549 cells were cultured on glass coverslips and infected with MVA+mycC16 at 5 PFU/cell. 5 h later, cells were fixed with 4% paraformaldehyde (PFA) for 15 min, permeabilized with 0.5% Triton X-100 for 15 min and blocked with 3% bovine serum albumin (BSA) in PBS for 30 min at room temperature. Primary antibodies were diluted in 3% BSA-PBS and incubated with cells at 4°C overnight. Samples were then washed with PBS, incubated with secondary antibodies conjugated with Alexa Fluor 488 or Alexa Fluor 594. Nuclei and virus factories were stained with DAPI and cells were washed multiple times prior to mounting on slides using ProLong Diamond Antifade reagent (ThermoFisher). Images were taken on a Leica SP8 confocal microscope and image processed with the Leica software (Leica Biosystems).

### Transmission electron microscopy

A549 cells and A549-ZAP-KO cells were infected with MVA at 10 PFU/cell. After 20 h, cells were fixed, dehydrated and embedded with Embed 812 resin (Electron Microscopy Sciences, Hatfield, PA as described [50]. Samples were imaged with an FEI Tecnai Spirit transmission electron microscope (FEI, Hillsboro, OR). Viral structures were manually counted on 50 cell sections.

## Supporting information

S1 Fig Expression and knockdown of ZAP in human cells. Indicated human cell lines in 24-well plates were transfected with 10 pM of a negative control siRNA (siNC) or two different sequence siRNAs to ZAP (siZ1 and siZ2) per well and harvested after 48 h. Total proteins were subjected to SDS-PAGE and ZAP was determined by Western blot analysis using antibodies to ZAP and GAPDH. The long (L) and short (S) forms of ZAP are labeled. The band migrating between L and S may be a cross-reacting protein. The numbers on the right indicate the electrophoretic positions of marker proteins in kDa.
(TIF)

S2 Fig Deletion of FAM111A does not rescue MVA replication in human cells. **(A)** Western blot of A549 and A549 ZAP/FAM111A DKO cells probed with antibodies to ZAP, FAM11A and GAPDH. **(B)** A549 or A549 ZAP-KO and A549 ZAP/FAM111A double knockout (DKO) cells were infected with RPXVΔC12 or MVA at 0.01 PFU/cell for 48 h and virus was titered on BS-C-1 cells by plaque assay. ** p<0.01; * p<0.05 by two-sided Student’s t-test.
(TIF)

S3 Fig Expression and stability of ZAP in MVA 51.2 infected cells. A549 cells were mock-infected or infected with MVA 51.2 at 4 PFU/cell. Total proteins from the cells were collected at 2, 4, 6 or 8 h post infection (h.p.i.) and analyzed by Western blotting with antibodies to ZAP, β-actin, viral early protein I3 and viral late protein A3.
(TIF)

S4 Fig Localization of C16 in A549 ZAP-KO cells. A549 or A549 ZAP-KO cells infected with MVA-2xMyc-C16 (MVA+C16) at 5 PFU/ cell for 5 h. Cells were then fixed, permeabilized, blocked and stained with primary antibodies to myc and ZAP followed by secondary fluorescent antibodies and DAPI to stain DNA.
(TIF)

S5 Fig Stress granule markers eIF4E and eIF4G do not colocalize with ZAP during infection. A549 cells were mock infected or infected with MVA-2xMyc-C16 (MVA+C16) at 5 PFU/ cell for 5 h. Cells were then fixed, permeabilized, blocked and stained with primary antibodies to eIF4E and ZAP (**A**) or eIF4G and ZAP (**B**) followed by fluorescent conjugated secondary antibodies. DAPI was used to stain DNA. Scale bar at bottom.
(TIF)

S6 Fig Relative abundances of MVA transcripts. RNAseq was carried out at 8 and 19 h after MVA infection of A549 and A549 ZAP-KO cells and analyzed as in Fig 5D except that the data were divided into transcripts of early, intermediate and late genes.
(TIF)

S7 Fig Expression and processing of viral proteins in MVA 47.1 infected cells. A549, A549 ZAP-KO cells stably transfected with C12 or an empty vector (vec) were mock infected or infected with MVA 47.1 and analyzed by Western blotting as for MVA in Fig 5G.
(TIF)

S1 Table Data set for human RNAi screen. (XLSX)

S2 Table Data set for RNAseq. (XLSX)

S3 Table Data set for mass spectrometry. (XLSX)
