# Supplementary figures and images for: Zinc-finger antiviral protein (ZAP) is a restriction factor for replication of modified vaccinia virus Ankara (MVA) in human cells
Source: PLoS Pathog. 2020 Aug 31;16(8):e1008845. doi: 10.1371/journal.ppat.1008845 (PMC7485971; doi:10.1371/journal.ppat.1008845)

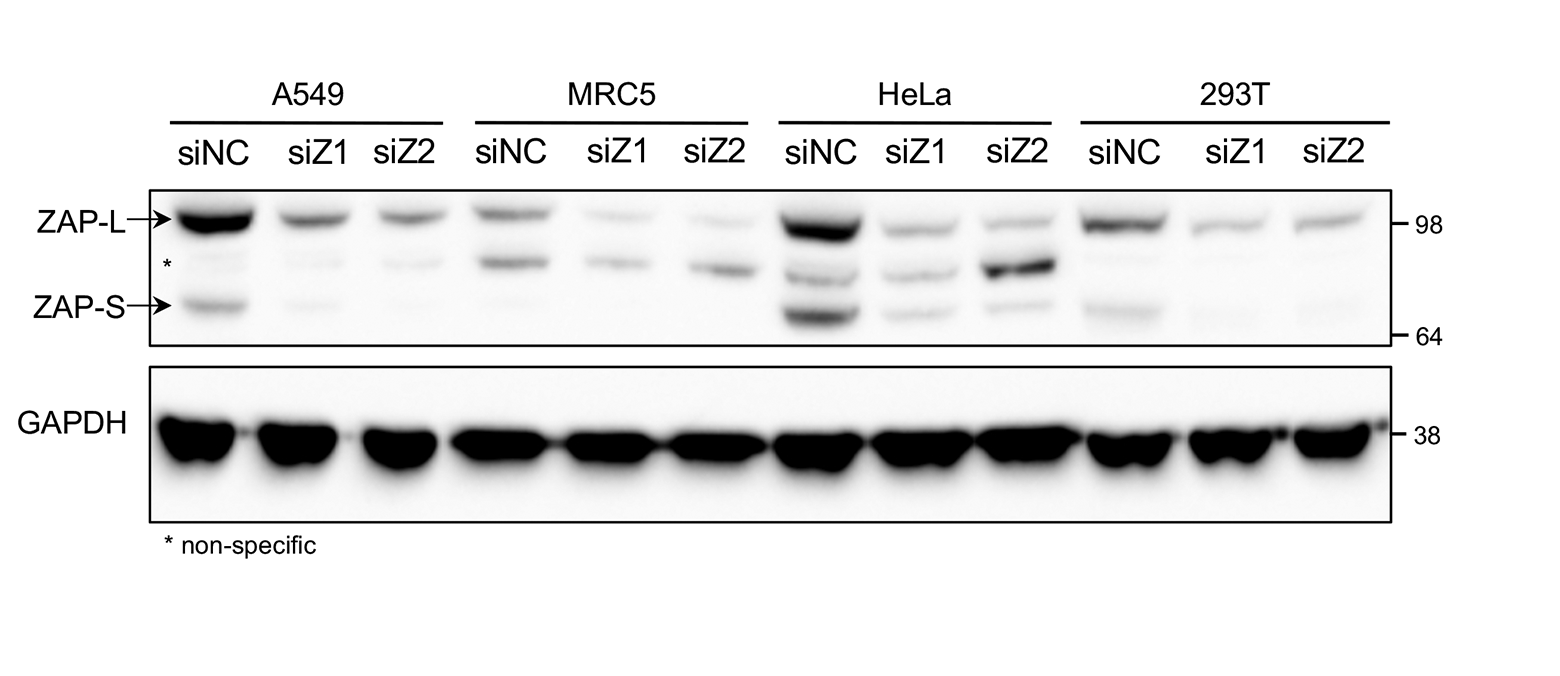

Supplement: S1 Fig — Indicated human cell lines in 24-well plates were transfected with 10 pM of a negative control siRNA (siNC) or two different sequence siRNAs to ZAP (siZ1 and siZ2) per well and harvested after 48 h. Total proteins were subjected to SDS-PAGE and ZAP was determined by Western blot analysis using antibodies to ZAP and GAPDH. The long (L) and short (S) forms of ZAP are labeled. The band migrating between L and S may be a cross-reacting protein. The numbers on the right indicate the electrophoretic positions of marker proteins in kDa. (TIF) [file ppat.1008845.s001.tif]

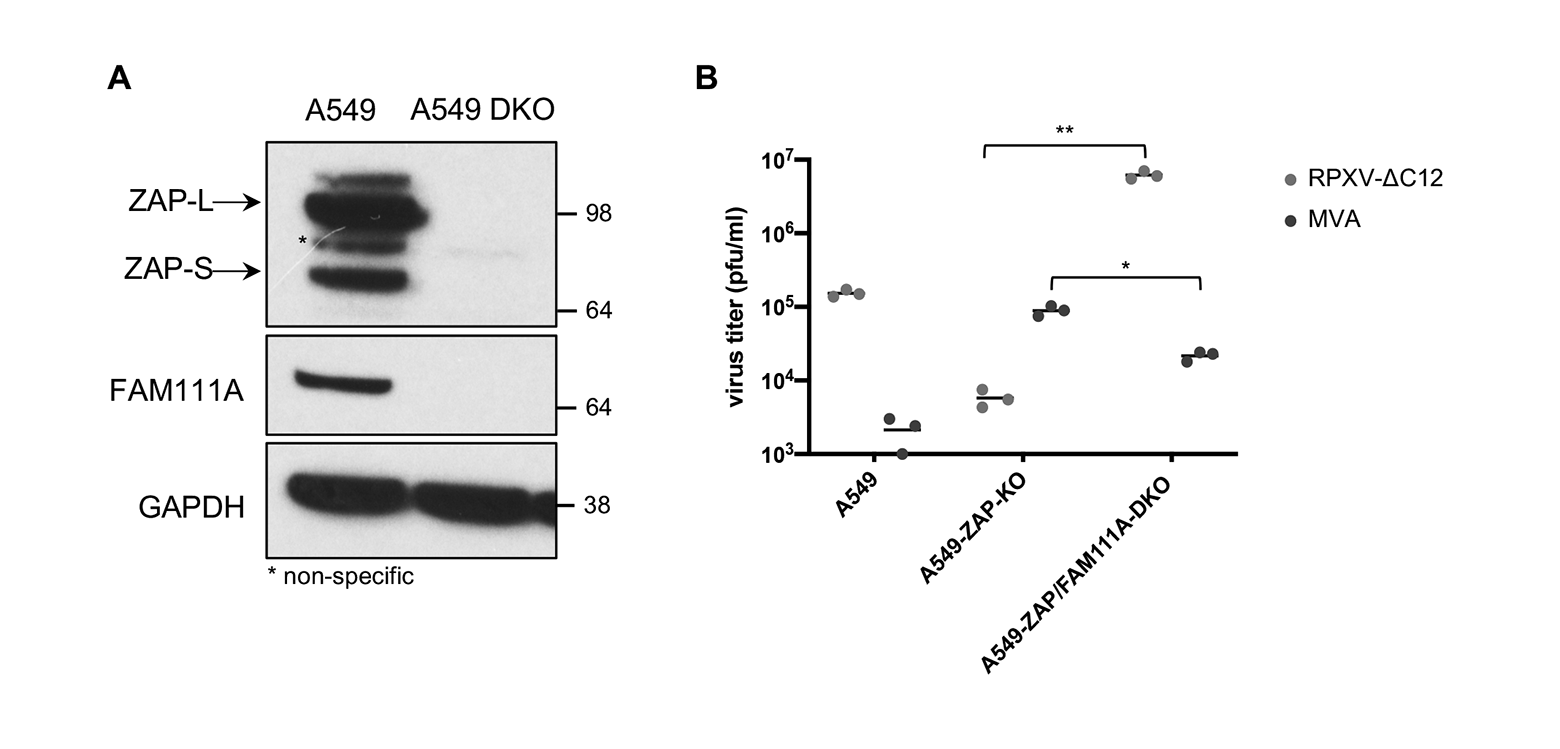

Supplement: S2 Fig — (A) Western blot of A549 and A549 ZAP/FAM111A DKO cells probed with antibodies to ZAP, FAM11A and GAPDH. (B) A549 or A549 ZAP-KO and A549 ZAP/FAM111A double knockout (DKO) cells were infected with RPXVΔC12 or MVA at 0.01 PFU/cell for 48 h and virus was titered on BS-C-1 cells by plaque assay. ** p<0.01; * p<0.05 by two-sided Student’s t-test. (TIF) [file ppat.1008845.s002.tif]

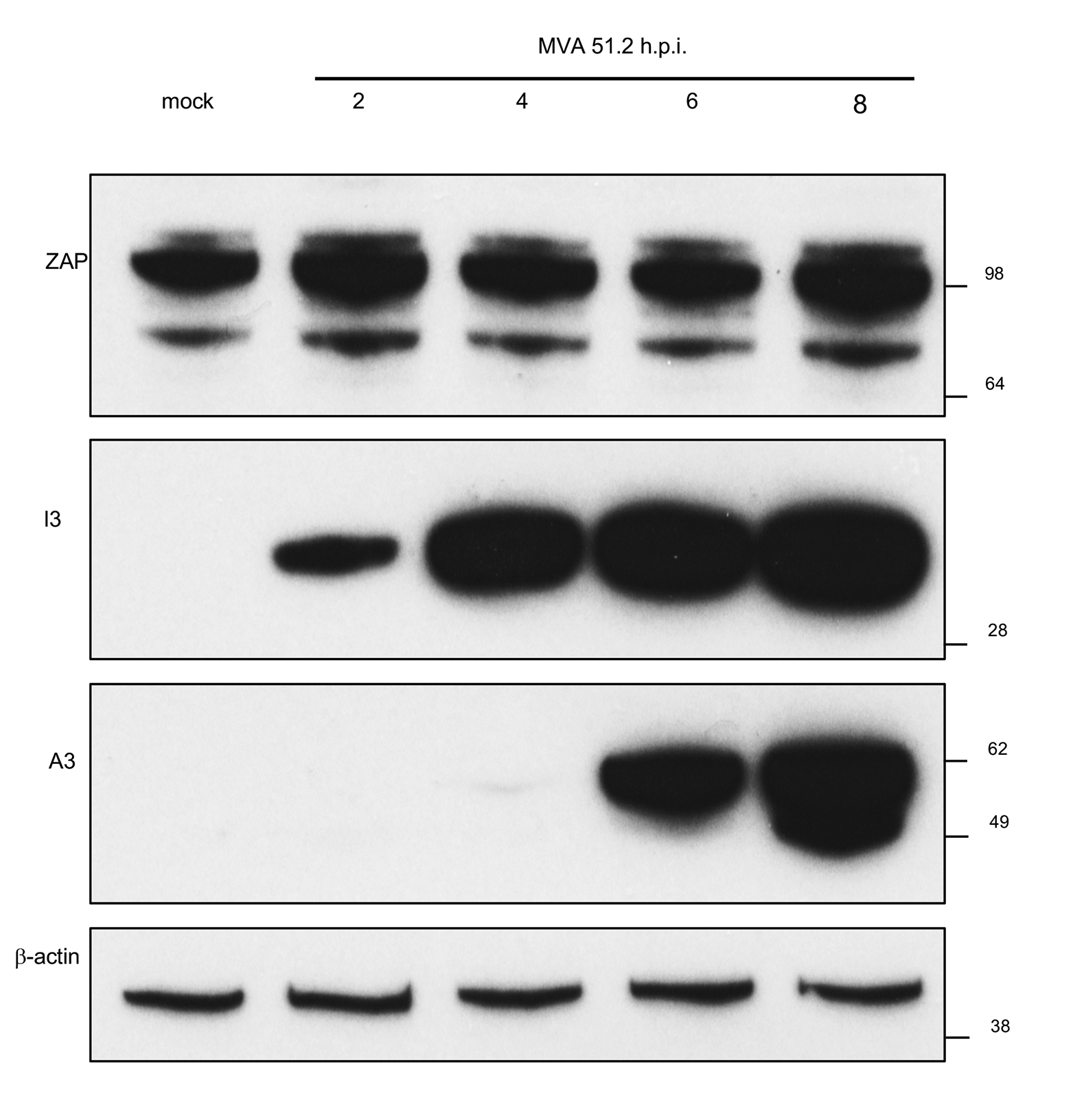

Supplement: S3 Fig — A549 cells were mock-infected or infected with MVA 51.2 at 4 PFU/cell. Total proteins from the cells were collected at 2, 4, 6 or 8 h post infection (h.p.i.) and analyzed by Western blotting with antibodies to ZAP, β-actin, viral early protein I3 and viral late protein A3. (TIF) [file ppat.1008845.s003.tif]

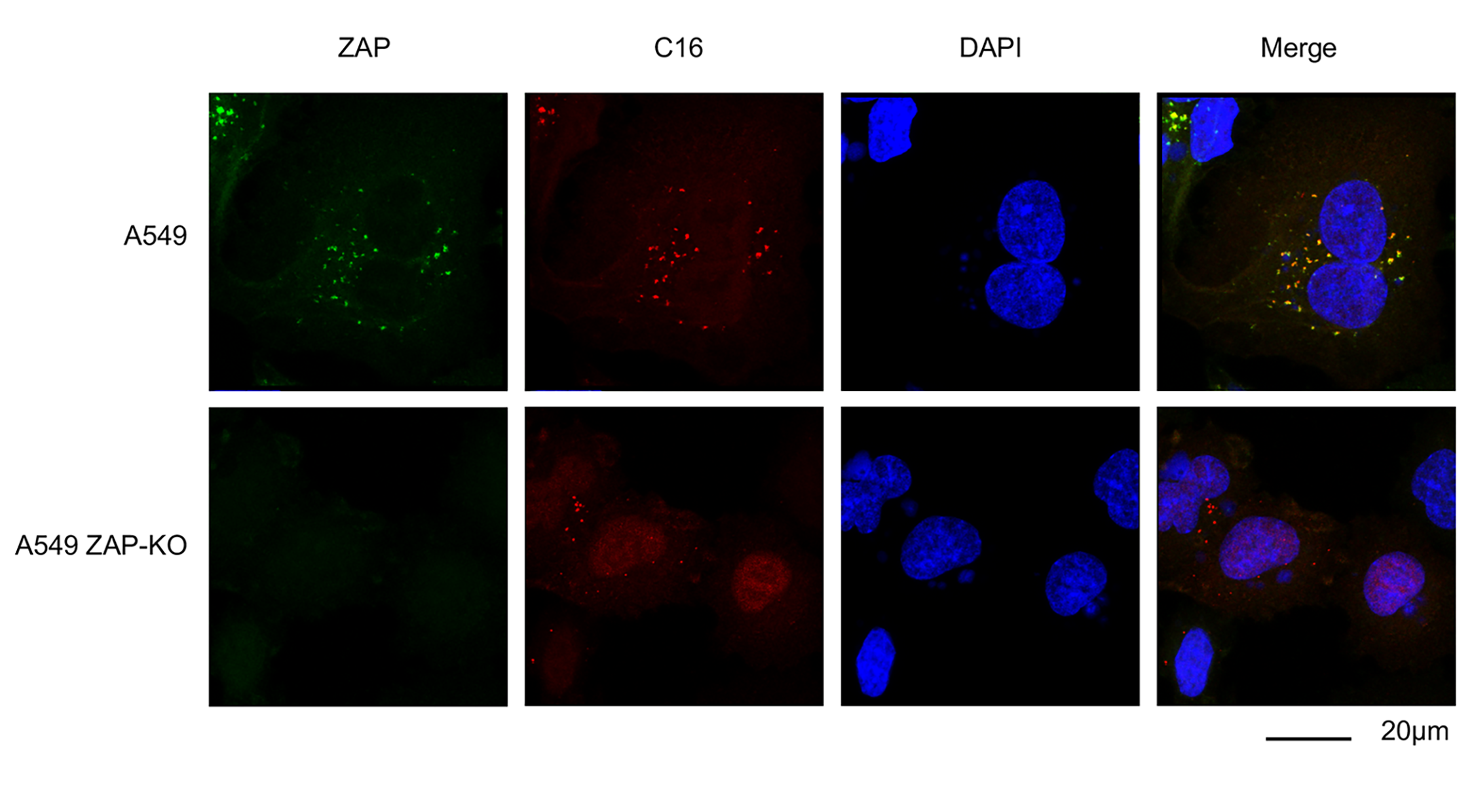

Supplement: S4 Fig — A549 or A549 ZAP-KO cells infected with MVA-2xMyc-C16 (MVA+C16) at 5 PFU/ cell for 5 h. Cells were then fixed, permeabilized, blocked and stained with primary antibodies to myc and ZAP followed by secondary fluorescent antibodies and DAPI to stain DNA. (TIF) [file ppat.1008845.s004.tif]

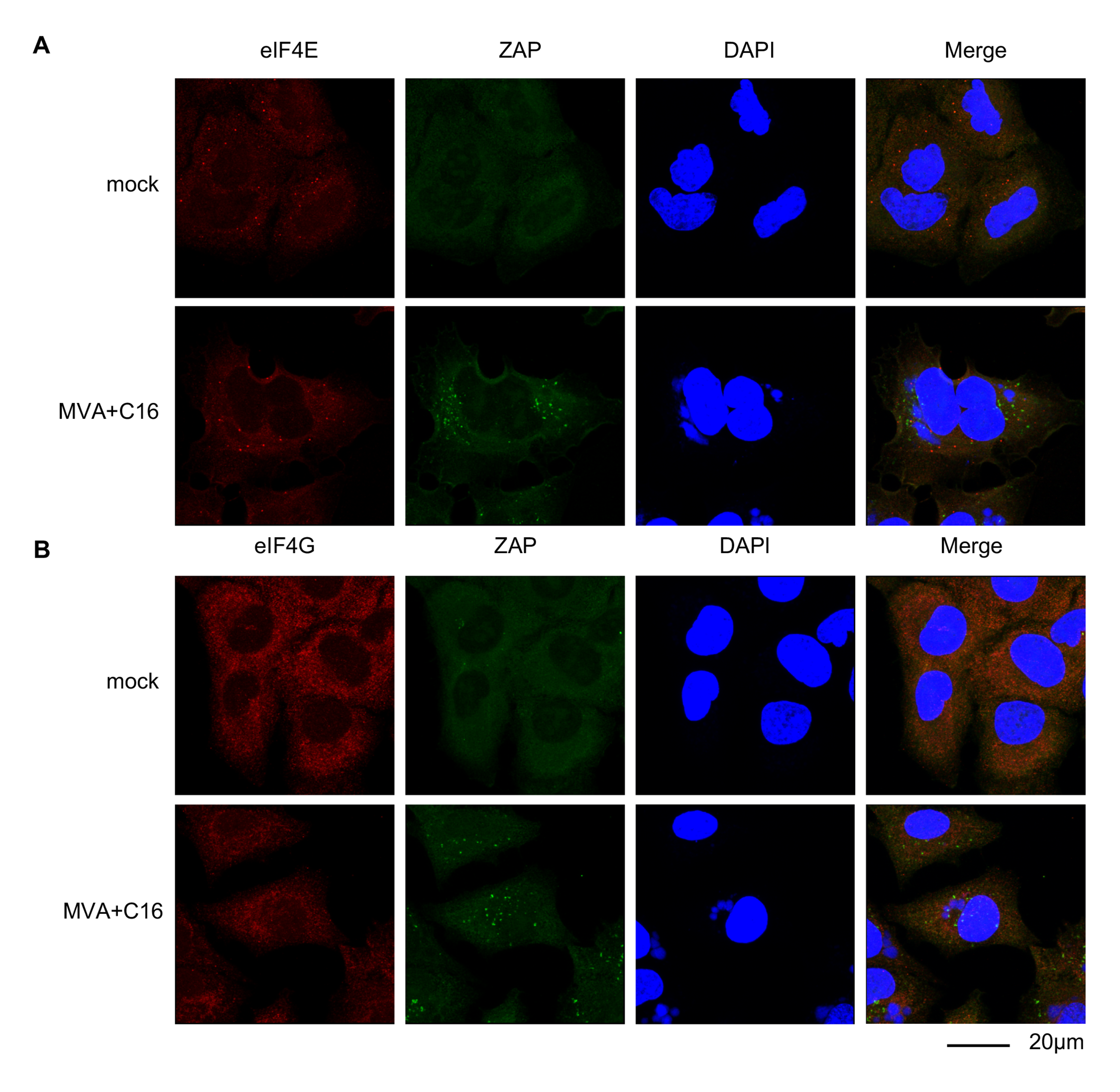

Supplement: S5 Fig — A549 cells were mock infected or infected with MVA-2xMyc-C16 (MVA+C16) at 5 PFU/ cell for 5 h. Cells were then fixed, permeabilized, blocked and stained with primary antibodies to eIF4E and ZAP (A) or eIF4G and ZAP (B) followed by fluorescent conjugated secondary antibodies. DAPI was used to stain DNA. Scale bar at bottom. (TIF) [file ppat.1008845.s005.tif]

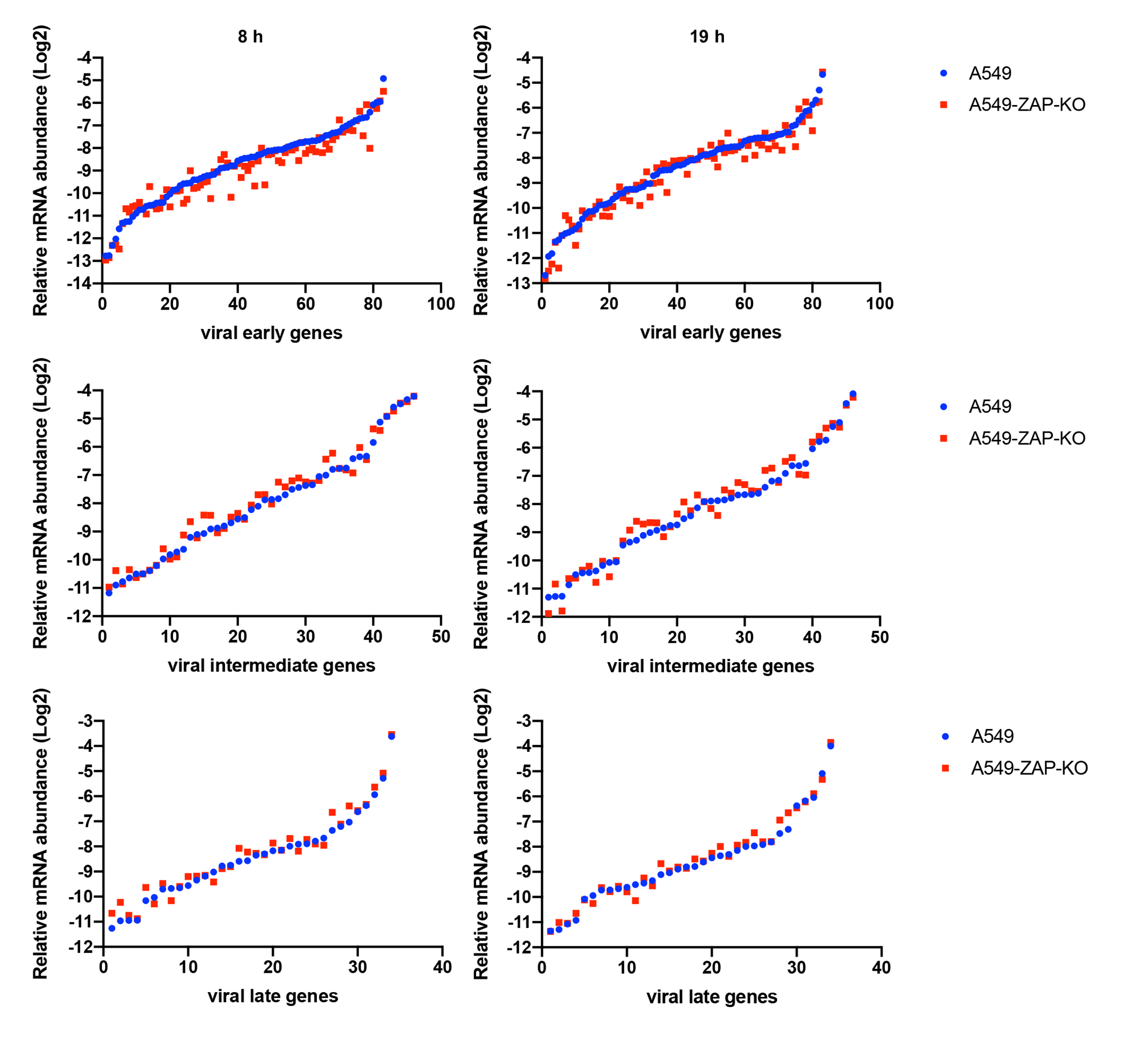

Supplement: S6 Fig — RNAseq was carried out at 8 and 19 h after MVA infection of A549 and A549 ZAP-KO cells and analyzed as in Fig 5D except that the data were divided into transcripts of early, intermediate and late genes. (TIF) [file ppat.1008845.s006.tif]

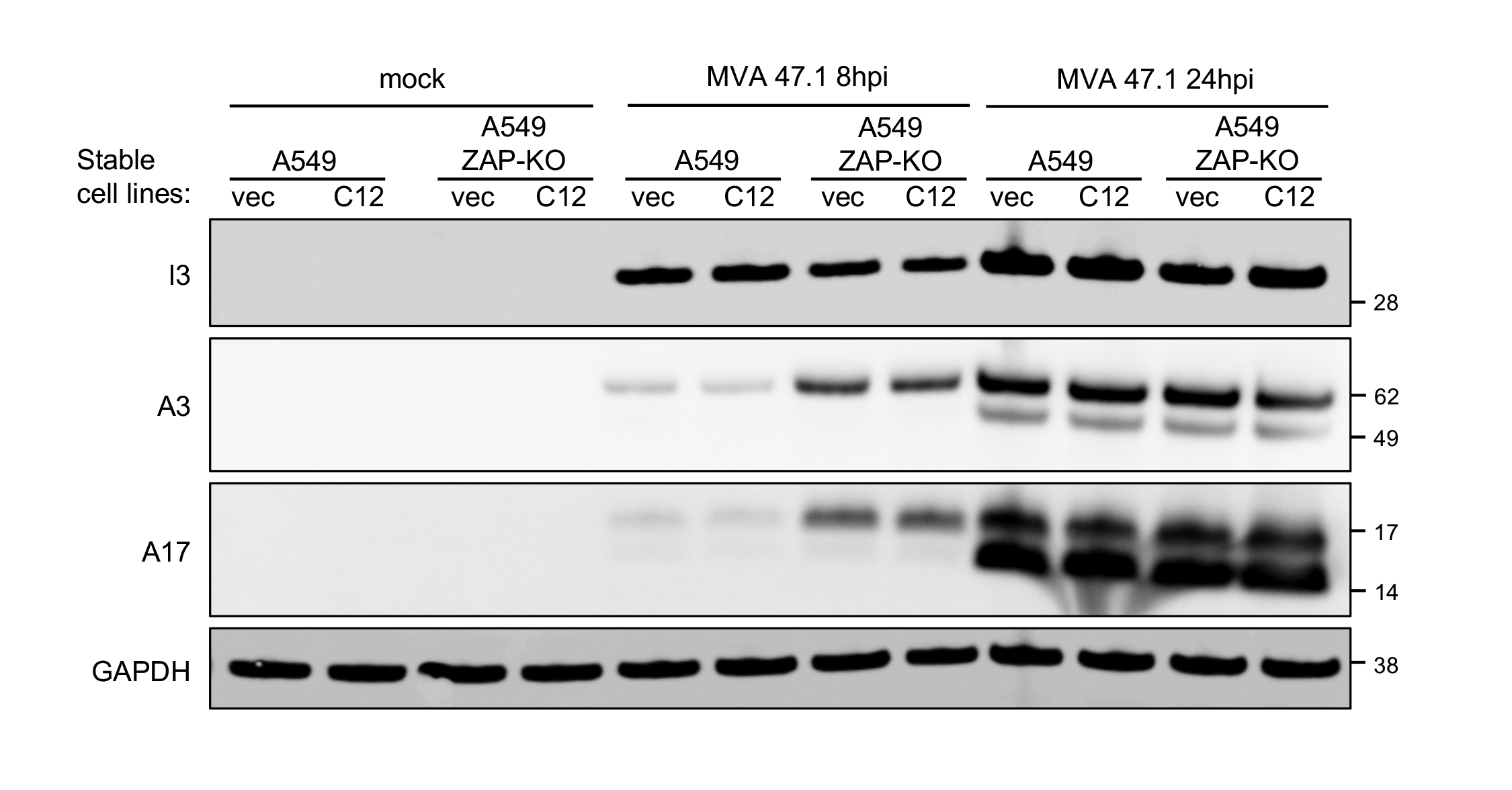

Supplement: S7 Fig — A549, A549 ZAP-KO cells stably transfected with C12 or an empty vector (vec) were mock infected or infected with MVA 47.1 and analyzed by Western blotting as for MVA in Fig 5G. (TIF) [file ppat.1008845.s007.tif]
